# Shifting focus: Time to look beyond the classic physiological adaptations associated with human heat acclimation

**DOI:** 10.1113/EP091207

**Published:** 2023-10-26

**Authors:** Thomas A. Deshayes, Dèwanou Gilles Arnaud Sodabi, Marianne Dubord, Daniel Gagnon

**Affiliations:** ^1^ Montreal Heart Institute Montréal Canada; ^2^ School of Kinesiology and Exercise Science Université de Montréal Montréal Canada

**Keywords:** cardiac, cognition, kidney, renal, sweat, temperature, thermoregulation

## Abstract

Planet Earth is warming at an unprecedented rate and our future is now assured to be shaped by the consequences of more frequent hot days and extreme heat. Humans will need to adapt both behaviorally and physiologically to thrive in a hotter climate. From a physiological perspective, countless studies have shown that human heat acclimation increases thermoeffector output (i.e., sweating and skin blood flow) and lowers cardiovascular strain (i.e., heart rate) during heat stress. However, the mechanisms mediating these adaptations remain understudied. Furthermore, several possible benefits of heat acclimation for other systems and functions involved in maintaining health and performance during heat stress remain to be elucidated. This review summarizes recent advances in human heat acclimation, with emphasis on recent studies that (1) advanced our understanding of the mechanisms mediating improved thermoeffector output and (2) investigated adaptations that go beyond those classically associated with heat acclimation. We highlight that these studies have contributed to a better understanding of the integrated physiological responses underlying human heat acclimation while leaving key unanswered questions that will need to be addressed in the future.

## Abstract (highlights)

- **What is the topic of this review?**
  This review summarizes studies that advance our understanding of the mechanisms mediating improved thermoeffector output following heat acclimation and investigate adaptations that go beyond those classically associated with heat acclimation.
- **What advances does it highlight?**
  Most studies that investigated possible mechanisms mediating greater thermoeffector output following heat acclimation have focused on end‐organ responses. Some evidence suggests that heat acclimation improves cardiac function, body fluid regulatory functions, and preserves cognitive performance under heat stress. However, these conclusions are based on limited and conflicting data.

## 1 INTRODUCTION

**FIGURE 1:**
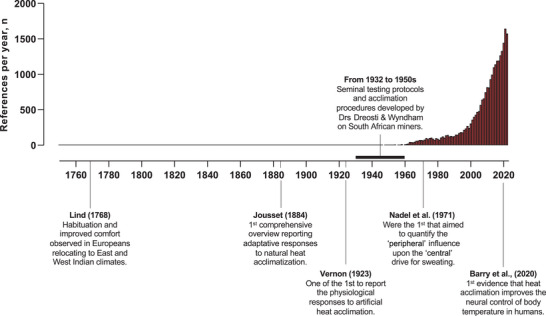
Change in the number of studies per year on heat acclimation/acclimatization from 1760 to 2022 showing some seminal references. See Periard et al. (2021), Ravanelli, Gendron et al. (2021), and Schneider (2016) for more details of historical review of human heat acclimation. The search was performed on August 2023 using PubMed (all fields, heat acclim* OR heat adapt*).

Mammals can adapt to a wide range of environmental stressors, which notably allowed humans to settle in regions with extreme climates. This was possible through genetic, cultural/behavioral, and functional (physiological) adaptations (Leonard, 2015). The latter are non‐genetic and occur during an individual's lifetime (i.e., phenotypic) if repeatedly exposed to acute stress (Leonard, 2015; Taylor, 2014). These acquired phenotypic adaptations to specified climatic components, which are termed acclimatization, improve the maintenance of homeostasis during subsequent exposures and, ultimately, reduce the inherent physiological strain. Although recognized for centuries (Jousset, 1884; Lind, 1768; Schneider, 2016; Vernon, 1923), human heat acclimation has attracted increased scientific interest in recent decades (Périard et al., 2015; Taylor, 2014) (Figure 1), particularly due to rising global temperatures.

Planet Earth is warming at an unprecedented rate and humans are already facing more frequent, intense, and long‐lasting episodes of extreme heat that will intensify in the coming years (Romanello et al., 2022). The 2023 northern hemisphere summer was marked by historic heat that affected vast areas of the USA, Europe, and Asia (CNN, 2023; Zachariah et al., 2023). At one time, over one‐third of the entire US population (>100 million people) were under a heat advisory (Reuters, 2023). The Secretary‐General of the UN warned that we have now entered a point of ‘global boiling’ (The Guardian, 2023; United Nations, 2023) and US President Biden announced unprecedented measures to protect the US population against heat extremes (The White House, 2023). More frequent and intense heat extremes will increasingly affect multiple facets of human life, including, but not limited to, activities of daily living, leisure and sports, and occupational activities. At the individual‐scale, the main impacts relate to health, safety and functioning (performance and productivity). Robust evidence has associated extreme heat with a greater risk of mortality across the globe (Gasparrini et al., 2017). In 2022, the hottest summer recorded to date in Europe caused over 60,000 heat‐related deaths (Ballester et al., 2023). Extreme heat is also associated with an increase in emergency room visits and hospitalizations (Cheng, Lung et al., 2019; Liss & Naumova, 2019; Onozuka & Hagihara, 2015), cardiorespiratory deaths (Cheng, Xu et al., 2019), kidney injuries and diseases (Liu et al., 2021), among others. Heat also impedes human functioning, affecting physical and cognitive performance (Martin et al., 2019), sleep quality (Rifkin et al., 2018), and mental health (Thompson et al., 2018), among others.

Heat acclimation is an effective strategy to improve the body's capacity to dissipate heat, increasing heat tolerance and lessening the risk of heat‐related injuries and performance decrements. These effects are generally attributed to adaptations in the thermoregulatory (Taylor, 2014) and cardiovascular (Périard et al., 2016) systems that have been widely observed and reported during natural (i.e., acclimatization; Brown et al., 2022) or artificial (i.e., acclimation; Périard et al., 2015) heat exposures. The first heat acclimation/acclimatization physiological experiments were performed at the beginning of the 20th century, with the seminal studies of Drs Dreosti and Wyndham evaluating South African miners in the 1930s–1940s (Schneider, 2016). These studies established what are now considered the classic adaptations induced by heat acclimation/acclimatization: a reduced resting core temperature, a reduced heart rate during physical work in hot conditions, and greater sweat output (Wyndham, 1967; Wyndham et al., 1968). Several studies have since confirmed these observations (see Périard et al. (2015) and Taylor (2014) for more details). In contrast, few studies have examined the possible physiological mechanisms that mediate these adaptations. Furthermore, other heat‐related adaptations have received less attention, even though they could be crucial for improving human health and functioning during heat stress (e.g., body fluid regulatory functions, cognitive performance). Therefore, in an effort to better understand the integrated physiological responses underlying heat acclimation, and to supplement previous ones, this review aims to summarize recent advances in this field. Our starting point was the comprehensive review by Taylor (2014), which allowed us to focus on subsequent studies that went beyond the classic heat‐related thermoeffector and cardiovascular adaptations. Furthermore, we aimed to highlight key unanswered questions to stimulate future research within this field.

## 2 HUMAN HEAT ACCLIMATION: BRIEF OVERVIEW OF METHODOLOGICAL ASPECTS

This section introduces the main concepts regarding heat acclimation/acclimatization that are needed to interpret the rest of the review. For further details, readers are referred to more comprehensive reviews (Périard et al., 2015; Taylor, 2014).

**FIGURE 2:**
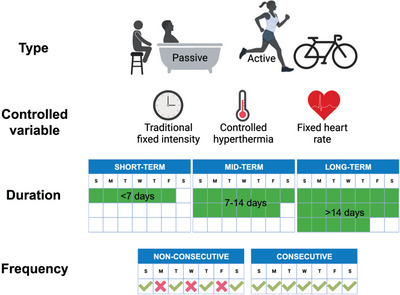
Parameters to consider when implementing heat acclimation protocols. Created with BioRender.com.

Heat stress, which depends on environmental conditions, metabolic heat production, and clothing, is the net heat load to which an individual is exposed (ACGIH, 2022). It contributes directly to homeostatic disturbances (i.e., heat strain) reflected in the physiological triad of hyperthermia, cardiovascular strain, and dehydration. Heat strain is therefore defined as the integrated physiological response resulting from heat stress (ACGIH, 2022). Heat acclimation/acclimatization is one of the various coping strategies that humans have developed in an attempt to minimize heat strain (Jay et al., 2021). It is well established that frequent exposures to heat that sufficiently induce homeostatic disturbances (i.e., increased core body temperature, skin blood flow, and sweat secretion) lead to physiological adaptations that, *in fine*, reduce subsequent heat strain and improve heat tolerance and resilience (Périard et al., 2015; Taylor, 2014). While they depend upon the magnitude of the thermal stress imposed on the body, as well as the number, the frequency, and the duration of heat exposures, a large proportion (∼75%) of the adaptations occur within 7 days (Périard et al., 2015). Figure 2 depicts the different parameters to consider when implementing heat acclimation protocols. First, heat acclimation may be induced actively, passively or a combination of both. Active approaches include exercising under hot conditions (i.e., active heat acclimation). Passive approaches include sauna bathing, environmental chamber exposure or hot water immersion. Combined approaches include, for example, exercising under cool conditions followed by passive heat exposure. All approaches can employ a fixed intensity/duration (i.e., traditional heat acclimation) or aim to clamp core body temperature (i.e., controlled hyperthermia) or heart rate. The choice of the approach depends on the available resources, the logistical constraints, the desire to be specific or not to the task that will have to be accomplished, and the population, among others (Ashworth et al., 2020; Periard et al., 2021). Protocols can also be of short (<7 days), medium (7–14 days) or long (>14 days) duration and the frequency of exposures can be consecutive (i.e., daily) or non‐consecutive (e.g., every 2 or 3 days).

The so‐called traditional acclimation model consists of the repeated application of heat stress of fixed intensity throughout exposures (i.e., constant forcing function). By applying a fixed external stimulus, heat strain (and thus the adaptation stimulus) decreases gradually as acclimation progresses. It has been suggested that this protocol is not optimal, leading to physiological habituation rather than adaptation per se (Taylor, 2014). The controlled hyperthermia model aims to keep the internal strain (and therefore the adaptation stimulus) constant, by maintaining a fixed internal temperature during each heat exposure (i.e., thermal clamping). This overcomes the habituation phenomenon observed during the traditional model (Taylor, 2014). However, to the best of our knowledge, only one study has directly compared both protocols during 5 and 10 days of heat acclimation, concluding that both protocols lead to similar adaptations, specifically a similarly decreased exercising heart rate and core body temperature and increased sweat losses (Gibson et al., 2015).

More recently, it has been proposed and discussed that heat acclimation can be similarly achieved using a constant forcing function by clamping heart rate rather than core body temperature (Périard et al., 2015, 2021; Taylor et al., 2020), which may be more applicable to field settings. Several studies have also examined whether heat acclimation with or without superimposed dehydration (2–3% of body mass) could affect the adaptation process and the inherent adaptations. For more details, readers are directed to the review by Sekiguchi et al. (2020). Briefly, with one exception (Travers, Nichols et al., 2020), these studies arrived at similar conclusions, showing that mild dehydration (2% of body mass) does not appear to alter the adaptations inherent to short‐ and medium‐term heat acclimation (Barley et al., 2020; Garrett et al., 2014; Haroutounian et al., 2021; Neal et al., 2016; Pethick et al., 2019; Schleh et al., 2018). The applicability of heat acclimation and its methods have developed gradually over the years. In its infancy, heat acclimation/acclimatization was mainly studied in occupational and military contexts, using medium‐ to long‐term consecutive and active (traditional model) heat exposures. Subsequently, interest in the impact of heat acclimation in the context of sports performance increased, which led to significant development of different methods of acclimation in terms of duration (short‐term) and methods (controlled hyperthermia and fixed heart rate).

## 3 IMPROVED THERMOEFFECTOR OUTPUT AND LOWER HEART RATE FOLLOWING HEAT ACCLIMATION – THE TIP OF THE ICEBERG

**FIGURE 3:**
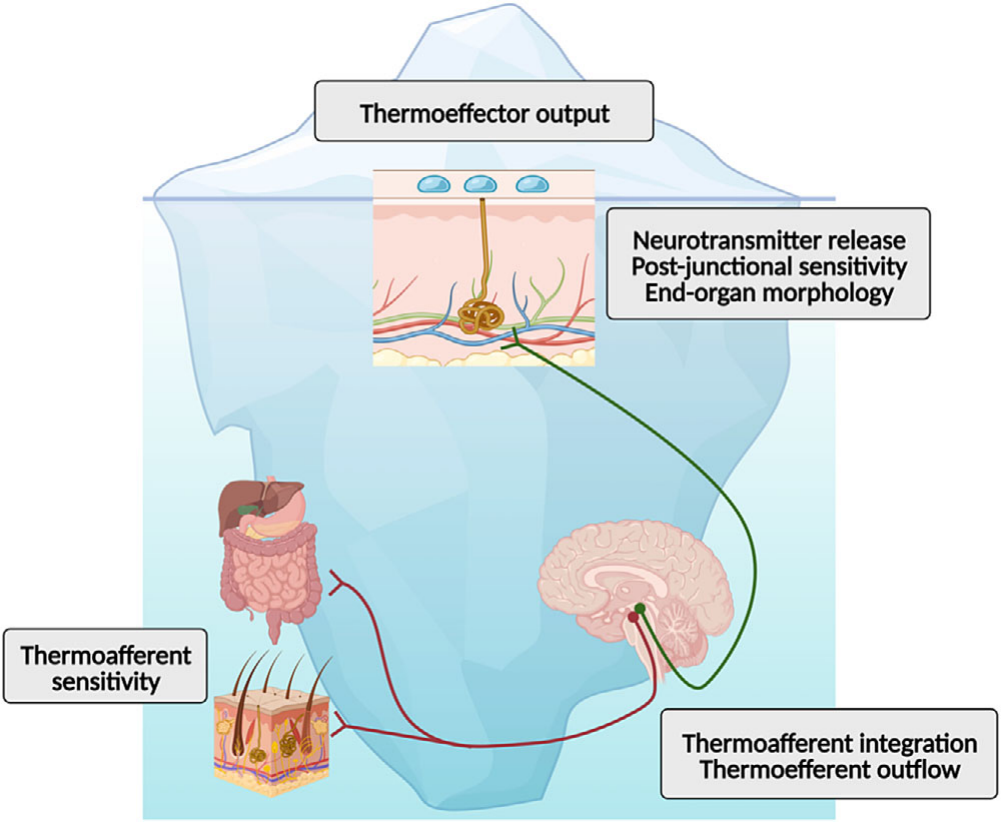
Improved thermoeffector output (e.g., increased sweat rate) is a hallmark of heat acclimation but it represents the end‐point (tip of the iceberg) of an underlying thermoregulatory reflex loop that has been understudied (beneath the water surface). Created with BioRender.com.

Thermoeffector output represents the endpoint of a sequence of coordinated autonomic responses that include thermoafferent sensing, central integration, thermoefferent signaling and end‐organ sensitivity (Cramer et al., 2022). Therefore, changes in thermoeffector output following heat acclimation can occur at any or multiple points along this thermoeffector loop, and thus represent the ‘tip of the iceberg’ of thermoregulatory adaptations in response to heat acclimation (Figure 3).

Improved thermoeffector output can originate from ‘neural’ (i.e., afferent signaling, integration, and efferent signaling) or ‘peripheral’ (i.e., end‐organ response) modulation (Ravanelli, Gendron et al., 2021). Most studies that investigated possible mechanisms mediating greater thermoeffector output following heat acclimation have focused on end‐organ responses, such as structural and functional changes in thermoeffector organs. Some studies provided evidence of structural adaptations, but only for the eccrine sweat gland. In patas monkeys, a greater length and volume of the secretory coil of isolated eccrine sweat glands was observed in response to continuous heat exposure (∼33°C, 13% relative humidity) for 9 weeks and 9 months (Sato et al., 1990). In humans, a greater sweat gland volume was observed in eccrine glands isolated from individuals who self‐reported being ‘heavy sweaters’ compared to individuals who self‐reported being ‘poor sweaters’ (Sato & Sato, 1983). Such structural changes may underlie functional adaptations of the sweat gland, as sweating of isolated eccrine glands in response to cholinergic stimulation correlates with sweat gland size (Sato & Sato, 1983; Sato et al., 1990). Indeed, studies have shown that heat acclimation improves the post‐junctional cholinergic sensitivity of sweat glands (Buono, Martha et al., 2009; Buono, Numan et al., 2009; Inoue et al., 1999; Lorenzo & Minson, 2010; Pearson et al., 2017), and also of the cutaneous microvasculature (Lorenzo & Minson, 2010).

In contrast, few studies have considered if heat acclimation improves the neural control of body temperature. To our knowledge, no studies have investigated if thermoafferent sensing or central integration of thermoafferent inputs are modified by heat acclimation. Although challenging, these remain exciting avenues for future research. For efferent signaling, the possibility that heat acclimation leads to greater thermoefferent outflow was directly tested by Barry et al. (2020) using microneurography. Barry et al. (2020) demonstrated that a 7‐day passive and controlled hyperthermia heat acclimation protocol lowers the mean body temperature onset threshold for the activation of skin sympathetic nerve activity during passive heat stress in young healthy males and females. There was also evidence of greater skin sympathetic nerve outflow during the initial phase of heat stress (increases in mean body temperature up to ∼1°C). The earlier activation of skin sympathetic nerve activity likely underlies the often‐observed faster activation of heat loss thermoeffectors following heat acclimation. Indeed, Barry et al. (2020) also observed a lower mean body temperature onset threshold for cutaneous vasodilatation and sweat production. A greater thermoefferent outflow may also lead to greater release of neurotransmitters and contribute to greater thermoeffector output following heat acclimation. Although this study established that heat acclimation improves the neural control of body temperature, it could not determine at what point along thermoeffector loops this improvement occurred. Multi‐unit skin sympathetic nerve activity measures neural activity directed to the cutaneous microvasculature and eccrine sweat glands. Therefore, the changes in skin sympathetic nerve activity observed could result from greater thermoafferent flow for a given thermal stimulus and/or a different central integration of thermoafferent inputs. Future studies are needed to investigate these possibilities.

The classic reduction in heart rate during heat stress that often accompanies heat acclimation has led to the hypothesis that cardiac function might be improved by heat acclimation (Périard et al., 2016; Taylor, 2014). This hypothesis is often substantiated with studies that employed rodent models and that demonstrated greater left ventricular compliance and reduced myocardial oxygen consumption in isolated cardiac tissue following heat acclimation (Horowitz et al., 1986; Mirit et al., 1999). However, these adaptations were observed in response to heat acclimation protocols that involved continuous exposure to a warm environment (34°C) for 1–2 months. It is also worth considering that the cardiovascular response to heat stress in rodents is markedly different from the one observed in humans. Heat stress causes a relatively large increase in mean arterial pressure (∼10–30 mmHg) in rodents (Kenney & Musch, 2004; Kenney et al., 1998), whereas mean arterial pressure does not change or decreases slightly (5–10 mmHg) in humans (Crandall & Wilson, 2015). This difference is likely explained by the fact that cutaneous vasodilatation is relatively minimal in rodents. Consequently, peripheral vascular resistance probably does not decrease when rodents are exposed to heat stress which contrasts with the robust reduction observed in humans (Gagnon et al., 2016; Minson et al., 1998; Rowell et al., 1969). Regardless, the substantial increase in arterial blood pressure likely provides a distinct stimulus for cardiac adaptation in rodents compared to humans. In fact, continuous exposure of rats to a warm environment (34°C, 40% relative humidity) for 1–2 months induces pathological remodeling of the heart, including myocardial tissue necrosis, fibrosis, and calcification (Yarom et al., 1990). It is noteworthy that these deleterious adaptations were noted in response to the same protocol that has been shown to elicit beneficial cardiac adaptations (Horowitz et al., 1986; Mirit et al., 1999). To our knowledge, no reason for these contradictory findings has been postulated.

**FIGURE 4:**
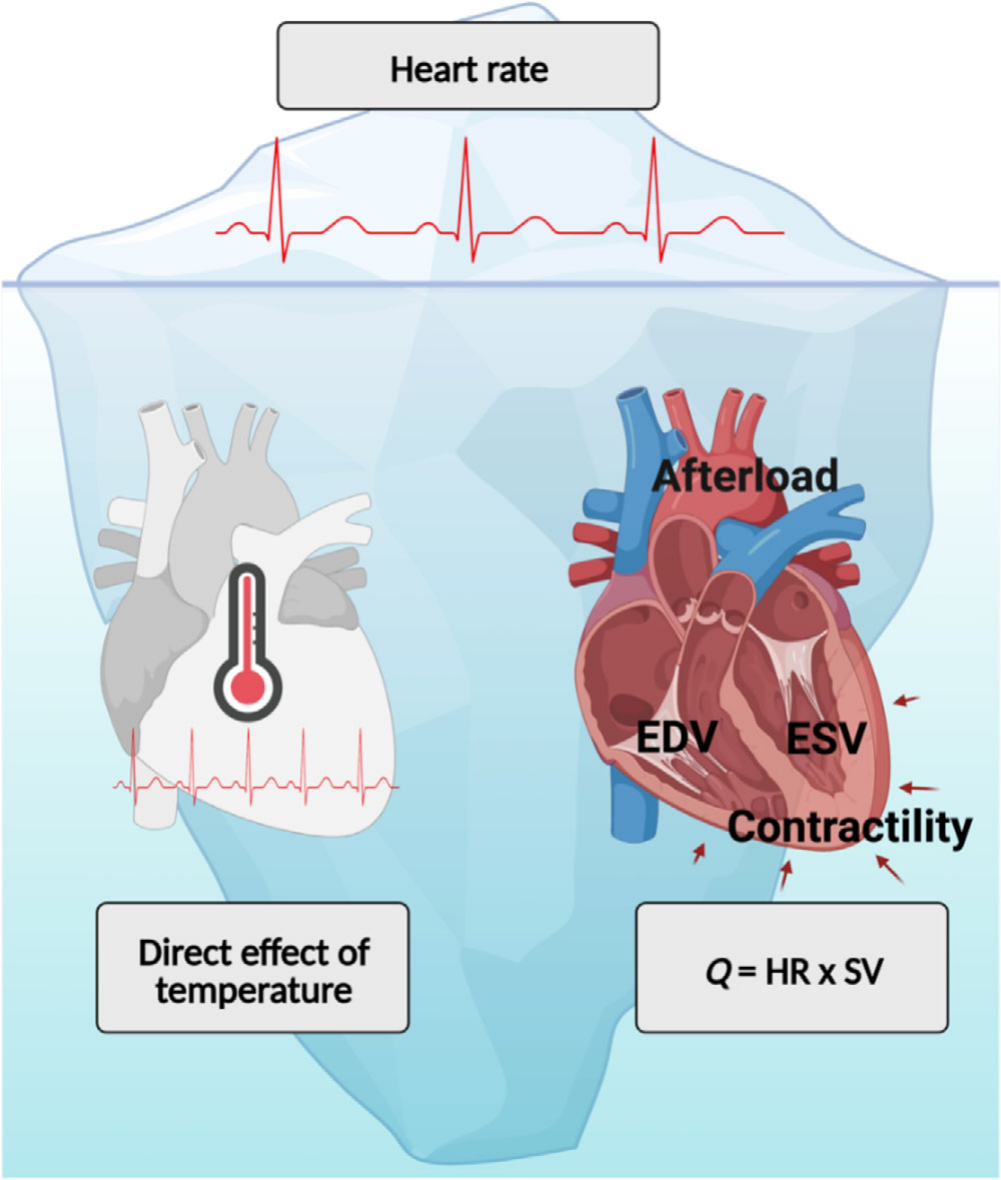
A reduced heart rate during heat stress is a hallmark of heat acclimation, but it represents the end‐point (tip of the iceberg) of underlying regulatory processes that have been understudied (beneath the water surface). EDV, end‐diastolic volume; ESV, end‐systolic volume; HR, heart rate; *Q*, cardiac output; SV, stroke volume. Created with BioRender.com.

Heart rate represents the end output of multiple regulatory processes, and thus represents the ‘tip of the iceberg’ of cardiovascular adaptations elicited by heat acclimation (Figure 4). The increase in heart rate during heat stress is primarily driven by autonomic neural adjustments and, to a lesser extent, the direct effect of temperature on the heart. The autonomic neural adjustments are best understood by the application of Ohm's law to the cardiovascular system, which states that mean arterial pressure is the product of cardiac output and peripheral vascular resistance. During passive heat stress, cutaneous vasodilation that is not compensated by vasoconstriction in other vascular beds (i.e., splanchnic, renal) results in a net reduction in peripheral vascular resistance (Gagnon et al., 2016; Minson et al., 1998; Rowell et al., 1969). Accordingly, cardiac output increases to defend arterial blood pressure, which remains relatively stable or decreases 5–10 mmHg during passive heat stress in the supine posture (Crandall & Wilson, 2015). The increase in cardiac output is primarily achieved via an increase in heart rate, as stroke volume remains stable (supine posture) or decreases (upright posture) during heat stress (Crandall & Wilson, 2015). Stroke volume itself is determined by: (i) cardiac preload, which decreases during heat stress (Gagnon et al., 2016; Wilson et al., 2009), (ii) cardiac contractility, which increases during heat stress (Gagnon et al., 2016; Wilson et al., 2009), and (iii) cardiac afterload, which remains relatively unchanged during heat stress (Gagnon et al., 2016; Wilson et al., 2009). The cardiovascular adjustments that occur during passive heat stress are generally amplified during exercise heat stress due to the superimposed requirements for blood flow to active skeletal muscles (Trangmar & González‐Alonso, 2019). It is also worth noting that heart rate influences stroke volume during exercise heat stress by limiting the time available for diastolic filling (Chou et al., 2019; Trinity et al., 2010). The direct effect of temperature on the heart stems from the effect of temperature on the sinoatrial (SA) and atrioventricular (AV) nodes. Specifically, increased temperature decreases action potential duration of the SA and AV nodes leading to a faster depolarization and therefore an increase in heart rate (Thibault et al., 1998; Yamagishi & Sano, 1967). During dual pharmacological blockade of cardiac parasympathetic (via atropine infusion) and sympathetic (via propranolol infusion) activity, heart rate increases ∼7 beats/min for each 1°C increase in mixed venous blood temperature during passive heat stress (Jose et al., 1970). Therefore, a reduction in heart rate during heat stress following heat acclimation could result from any or several of these regulatory processes.

When heat acclimation involves fixed‐intensity exercise (traditional models) and/or is achieved passively, cardiac output and blood pressure during heat stress remain relatively stable over the course of the acclimation period (Nielsen et al., 1993; Pallubinsky et al., 2017; Rowell et al., 1967; Trachsel et al., 2020; Wyndham et al., 1968, 1976). Accordingly, a reduced heart rate can be attributed to changes in stroke volume and/or a reduced direct effect of temperature on cardiac pacemaker cells. In terms of stroke volume, initial studies observed that heat acclimation minimizes the reduction in stroke volume that accompanies exercise heat stress (Nielsen et al., 1993; Rowell et al., 1967; Wyndham et al., 1968, 1976). This response was attributed to plasma volume expansion and a presumed increase in cardiac preload (Senay et al., 1976). However, core and skin temperatures during exercise heat stress were nearly always reduced as acclimation progressed in these early studies. Presumably, this lower thermal strain may have resulted in less of a direct effect of temperature on cardiac pacemaker cells and/or less skin blood flow requirements. This was nicely summarized by Rowell et al. (1967) who concluded that the cardiovascular system may be operating in a ‘cooler functional environment’ following heat acclimation. Nonetheless, some studies observed a lower heart rate during heat stress following heat acclimation without accompanying reductions in thermal strain (Convertino et al., 1980; Nielsen et al., 1993, 1997; Patterson et al., 2014; Racinais et al., 2017). Such observations provide stronger evidence that a lower heart rate may reflect improved cardiac function. It was not until the late 2010s that studies began investigating this possibility with echocardiography.

In response to a 12‐day passive and traditional heat acclimation protocol, Wilson et al. (2020) observed a greater normothermic and resting left ventricular end‐diastolic volume and greater early diastolic mitral annular tissue velocity (*E*′), an index of cardiac diastolic function, in young healthy males. These observations were later corroborated by Parsons et al. (2020) who also observed a greater normothermic and resting left ventricular end‐diastolic volume and early diastolic filling velocity, in addition to a greater stroke volume and left atrial volume after a 5‐day active and controlled hyperthermia heat acclimation protocol in young healthy males. However, these findings were not corroborated by two other studies that also investigated if heat acclimation alters echocardiography makers of cardiac function during heat stress. First, Trachsel et al. (2020) did not observe any changes in normothermic and resting cardiac volumes or markers of cardiac systolic or diastolic function following a 7‐day passive and controlled hyperthermia heat acclimation protocol in healthy young males and females. During subsequent passive heat stress, an attenuated decrease in left ventricular end‐diastolic volume, a greater atrial contribution to diastolic filling, and an attenuated increase in left atrial strain was observed following heat acclimation. These observations are consistent with improved cardiac diastolic function during heat stress following heat acclimation. Importantly, these changes occurred in the presence of a fixed thermal stimulus between the pre‐ and post‐acclimation visits. However, heart rate, stroke volume, and cardiac output during heat stress did not differ throughout heat acclimation. As such, the functional importance of the changes in diastolic function observed remains unclear. Second, Travers, González‐Alonso et al. (2020) investigated possible changes in cardiac function during exercise heat stress in response to a 10‐day active and controlled (fixed heart rate) heat acclimation protocol in young healthy males. Following heat acclimation, a greater stroke volume was observed under normothermic and resting conditions, but none of the markers of cardiac function measured were altered. During upright exercise heat stress, a lower heart rate (∼7 bpm) was observed post‐acclimation albeit this was accompanied by a slightly lower core temperature (∼0.2°C). In contrast, no reduction in heart rate was observed post‐acclimation during semi‐recumbent exercise heat stress that was performed to obtain echocardiographic measures of cardiac function. There were also no changes in any of the markers of cardiac function measured during exercise. Travers et al. (2021) subsequently demonstrated that the addition of mild dehydration (∼3% body mass loss) during the heat acclimation protocol did not alter any of these results.

Taken together, these studies provide limited evidence that heat acclimation elicits adaptations in cardiac function under normothermic rest and passive or active heat stress. However, there are important limitations to these studies that are worth considering when interpreting these findings. First, the improvements observed under resting normothermic conditions may reflect plasma volume expansion that increases left ventricular end‐diastolic volume and therefore cardiac preload. The studies by Trachsel et al. (2020) and Travers, González‐Alonso et al. (2020) did not observe evidence of plasma volume expansion in response to heat acclimation. This may explain the limited (Trachsel et al., 2020) or absent (Travers, González‐Alonso et al., 2020) adaptations in cardiac function during heat stress in these studies. Second, no reduction in resting core temperature was observed in response to the heat acclimation protocol employed by Travers, González‐Alonso et al. (2020). This raises the possibility that the protocol did not elicit heat acclimation. A greater sweat rate was observed during the heat acclimation period, but exercise intensity increased progressively to maintain a fixed heart rate. As such, the greater sweat rate may have been due to greater metabolic heat production rather than improved sweating capacity. Third, Trachsel et al. (2020) measured cardiac function during passive heat stress in the supine position. It is possible that the absence of orthostatic stress may have masked any additional adaptations of cardiac function caused by heat acclimation. Indeed, Travers, González‐Alonso et al. (2020) only observed a lower heart rate after heat acclimation when exercise heat stress was performed upright. This reduction in heart rate was not observed when exercise heat stress was performed in the semi‐recumbent position. Taken together, it remains to be determined if cardiac function during heat stress is improved in response to heat acclimation, beyond improvements related to possible plasma volume expansion and especially during combined heat and orthostatic stress, which is more realistic of daily activities. Furthermore, it is important to highlight that few studies have considered if heat acclimation alters blood flow and/or volume redistribution during heat stress and how this may or may not affect peripheral vascular resistance.

## 4 HUMAN HEAT ADAPTATION BEYOND THERMOEFFECTOR OUTPUT AND HEART RATE

**FIGURE 5:**
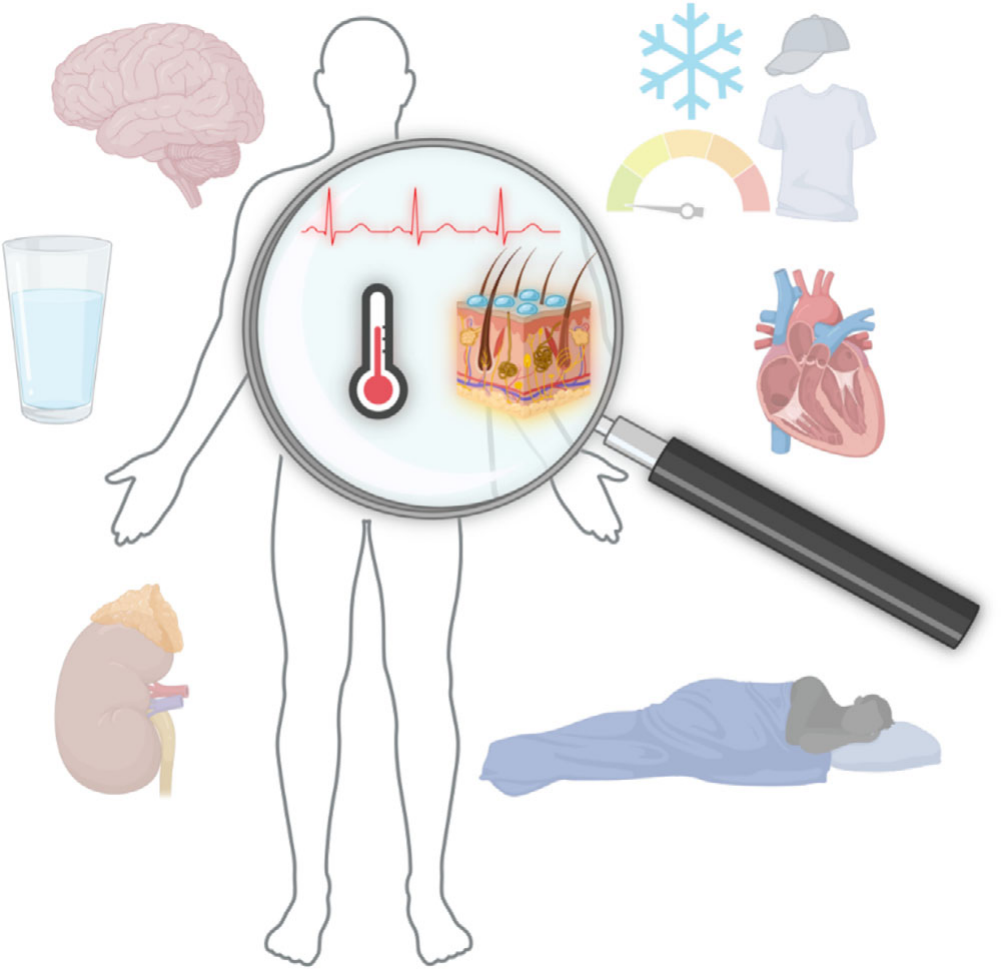
Schematic representation showing the three most studied variables in the field of heat acclimation (inside the magnifying glass) compared to some examples of other understudied variables (outside the magnifying glass) that are affected by heat stress. Created with BioRender.com.

It is obvious that the repercussions of heat are not limited to body hyperthermia and greater cardiovascular strain. For example, heat stress has a harmful effect on cognitive functions (Martin et al., 2019), which may lead to occupational accidents and traumatic injuries (Calkins et al., 2019; Fatima et al., 2021; Lee et al., 2019; Spector et al., 2016). In addition, heat poses a significant stress on water–electrolyte balance and kidney function. Evidence indicates that fluid and electrolyte disturbances and kidney injuries are among the main causes of hospital admittance during extreme heat events (Bobb et al., 2014; Hansen et al., 2008). Although much of the heat acclimation literature has focused on thermoeffector output, heart rate, and physical performance, some studies have gone further, looking at possible effects on cognitive performance and body fluid regulatory functions (Figure 5). The following section summarizes these studies.

### 4.1 Cognitive functions

The effect of acute heat stress on cognitive functions has largely been studied in the past five decades. It is generally observed that heat‐induced cognitive impairment mainly depends upon the magnitude of thermal (skin and core temperatures) and perceptual (thermal comfort) strain experienced (Gaoua et al., 2012), as well as the complexity of the task to be accomplished (Martin et al., 2019; Taylor et al., 2015). The relationship between core temperature and cognitive performance is not linear, but rather follows an inverted U‐shape (Schmit et al., 2017) with (i) an initial positive effect of heat stress on simple (i.e., basic mental processes requiring little conscious effort to perform, e.g., reaction time) and complex (i.e., heavy/complex mental operations such as memory, reasoning, decision making, e.g., tasks of executive function) cognitive processes with elevations in core temperature up to ∼38.5°C; (ii) a plateau of heat‐induced cognitive improvements beyond this threshold; and (iii) a cognitive performance decrement when core temperature exceeds ∼39.0°C, depending on task complexity with complex processes the first to be impaired. Since heat acclimation reduces thermal and perceptual strain, it has been investigated as a possible strategy to attenuate heat‐induced cognitive impairments (Gaoua et al., 2012; Schmit et al., 2017).

Shvartz et al. (1976) were the first to report faster reaction times during exercise under hot conditions (40°C, 48% relative humidity) after 8 days of active heat acclimation in healthy males. Walker et al. (2001) also reported improvements during simple psychomotor tests during passive heat stress after 4 days of passive heat acclimation in healthy males. Radakovic et al. (2007) observed decreased accuracy during a rapid visual information processing test immediately after an exertional heat stress test in unacclimated participants, but not in participants who previously underwent 10 days of passive or active heat acclimation. Tamm et al. (2015) reported that 10 days of active heat acclimation improves temporal processing (i.e., the subjective perception of time during exercise). Nonetheless, it is noteworthy that all these studies used traditional heat acclimation protocols, during which improved cognitive performance occurred alongside a progressive reduction in heat strain during heat acclimation (e.g., reduced heart rate and core temperature, lowered perceived thermal strain). Although this effect is desirable from a practical point of view (e.g., occupational settings), it is unclear whether heat acclimation improves cognitive performance independently of reductions in thermal and/or perceptual strain. Studies that compared cognitive performance before and after heat acclimation at fixed levels of heat strain generally do not report an effect of heat acclimation on cognitive performance. Of these studies, only Racinais et al. (2017) reported that acute heat‐induced impairments in executive function were reversed after 10 days of passive and traditional heat acclimation. No effect was observed on repeated acquisition task performance (Curley & Hawkins, 1983), visual attention, temporal and spatial orientation, and visual perception (Patterson et al., 1998) and complex cognitive and motor task performance (Piil et al., 2019) after active and traditional (Curley & Hawkins, 1983; Piil et al., 2019) or active and controlled (Patterson et al., 1998) heat acclimation protocols ranging between 10 and 28 days. Recently, Barry et al. (2022) reported no effect of a 7‐day passive and controlled heat acclimation protocol on processing speed or executive functions during heat stress. Nonetheless, an important limitation of the studies performed by Patterson et al. (1998) and Barry et al. (2022) is that no acute heat‐induced impairment in cognitive performance was observed pre‐acclimation, affecting the ability to determine the effects of subsequent heat acclimation.

Various methodological aspects likely contribute to the variability observed between studies, including but not limited to the cognitive tasks (e.g., domains assessed, tests used, complexity of the task), the acclimation protocol (mode, duration, frequency, confounding effect of exercise on cognitive functions) and the study design (lack of familiarization, population studied). It should also be noted that among the 123 participants of these nine studies, only four were females (3%). Furthermore, most studies recruited young (≤40 years old) healthy, and often trained participants, which reduces the generalizability of these data to the general population. Taken together, it is difficult to determine if heat acclimation represents an effective strategy to minimize the detrimental effects of heat stress on cognitive performance, when they occur. More efforts are needed to pursue our understanding of the role that heat acclimation may have in mitigating the adverse effects of heat on cognitive functioning, as recently highlighted by Donnan et al. (2023).

### 4.2 Body fluid regulation

Heat acclimation has been shown to improve some components of body fluid regulation, such as increased total body water, greater plasma volume, increased resting aldosterone concentrations, and reduced sodium sweat losses (Périard et al., 2015; Tyler et al., 2016). However, there is limited evidence about its impact on fluid acquisition (e.g., voluntary fluid intake) and thus water balance (e.g., voluntary dehydration) during heat stress. There is also limited evidence on the effects of heat acclimation on kidney function, which plays a key role in the regulation of electrolytes and water homeostasis. Such a strategy may also lessen the risk of heat‐related kidney injury/disease.

During passive or active heat stress, individuals replace approximately half of their fluid losses when drinking according to their thirst or ad libitum, although important intra‐individual variability exists (Cheuvront & Haymes, 2001; Goulet & Hoffman, 2019). This phenomenon, initially brought to light by the work of Adolph (1947), is known as voluntary dehydration. The progressive increase in sweat rate that occurs during heat acclimation increases the magnitude of voluntary dehydration if fluid intake does not increase proportionally. Eichna et al. (1945) and Greenleaf et al. (1983) reported an increase in voluntary fluid intake that overcompensated the increase in sweat rate as heat acclimation progressed, leading to a reduction in voluntary dehydration over 8–10 days of active and traditional heat acclimation in young healthy males. Interestingly, greater voluntary fluid intake appeared to be driven by a change in fluid intake pattern, with a shortened time to first drink, increased number of drinks consumed, and greater volume per drink (Greenleaf et al., 1983; unpublished observations reported in Hubbard et al., 1990). Based on these findings, it has been frequently argued that heat acclimation reduces voluntary dehydration by ∼30% during exercise heat stress (Périard et al., 2015, 2016). However, this argument contrasts sharply with the small and non‐significant decrease in voluntary fluid intake observed in the meta‐analysis by Tyler et al. (2016).

A closer look at the literature and recent studies suggests that heat acclimation is not always accompanied by increased voluntary fluid intake. Ormerod et al. (2003) observed a greater time to first drink alongside a reduced number of drinks consumed, and consequently a decreased overall fluid intake over 6 weeks of active and traditional heat acclimation in healthy females. However, the participants alternated between active heat exposures and winter outdoor training sessions, which may have reduced the effectiveness of the heat acclimation protocol. Furthermore, sweat rate was not measured during the exercise sessions which precludes knowing how heat acclimation impacted voluntary fluid replacement and thus voluntary dehydration. Other studies have observed that voluntary fluid intake increases but merely offsets greater sweat losses, thereby resulting in no net change in the extent of water balance deficit over 6–12 days of active and traditional heat acclimation in healthy young males (Greenleaf et al., 1967; Strydom et al., 1966), 5 days of active and controlled hyperthermia heat acclimation in healthy young males (Sekiguchi et al., 2021), or 7 days of passive and controlled hyperthermia heat acclimation in healthy young males and females (Ravanelli, Barry et al., 2021). For example, Pryor et al. (2020) observed that young healthy males replace 68% of fluid losses during a 4‐h intermittent exercise protocol (40°C, 40% relative humidity) after 4 days of active and controlled hyperthermia heat acclimation compared with 69% pre‐acclimation. Such observations are indirectly supported by studies that observed no reduction in the percentage of body mass loss during heat acclimation when drinking ad libitum (Bean & Eichna, 1943; Racinais et al., 2015; Sunderland et al., 2008; Yeargin et al., 2006). Interestingly, Strydom et al. (1966) found that the level of voluntary dehydration of males drinking ad libitum during heat acclimation was reduced from 1 litre after the first exposure to 0.3–0.5 litre after the third to fifth exposure. However, voluntary dehydration returned to 1–1.3 litres by the 10th–12th exposure (i.e., a U‐shape curve). This suggests that the duration of the acclimation protocol could, in part, influence the observed effect on voluntary fluid replacement. Furthermore, voluntary dehydration and its evolution during the development of acclimation could be modulated by age. Zappe et al. (1996) observed that plasma volume was increased in young (24 ± 2 years old) but not older (67 ± 1 years old) healthy males after 4 consecutive days of active and traditional heat acclimation. The increased plasma volume was associated with greater fluid intake during exercise in young compared with older males. Takamata et al. (1999) replicated these observations, showing that 6 days of active and traditional heat acclimation increase plasma volume in young (25 ± 3 years old) but not older (70 ± 3 years old) healthy males. They also observed a reduction in voluntary dehydration after heat acclimation in young but not older males during a 2‐h rehydration period that followed an acute dehydration of ∼1–2% of body mass induced by intermittent light exercise.

Since voluntary water intake is strongly influenced, but not exclusively, by thirst, some studies have focused on its evolution as heat acclimation develops. Some studies have reported no change (Sunderland et al., 2008) or a slightly reduced (Ormerod et al., 2003; Sekiguchi et al., 2021; Yeargin et al., 2006) thirst perception after heat acclimation, which was emphasized in the meta‐analysis by Tyler et al. (2016). However, the sensation of thirst is affected by several factors, including plasma osmolality and plasma volume changes, as well as anticipatory factors. Ingestion of water also rapidly decreases the feeling of thirst through the oropharyngeal reflex. These studies generally did not control for these factors. It is therefore difficult to explain/interpret these results and further studies are needed. For example, it would be interesting to know how heat acclimation alters the perception of thirst for a given increase in plasma osmolality or reduction in plasma volume. Further attention to this question could, for example, allow (i) refining the recommendations for hydration during exercise in the heat, which to date do not take into account the status of acclimation (Sekiguchi et al., 2021), (ii) understanding by what mechanisms heat acclimation might reduce the negative effect of heat on human performance, since greater thirst has been shown to exacerbate mental fatigue (Goodman & Marino, 2021; Suh et al., 2021), which may affect physical (Sawka & Noakes, 2007) and cognitive (Edmonds et al., 2013) performance.

The impacts of dehydration/hypohydration on endurance (Merry et al., 2010), muscle (Savoie et al., 2015), and cognitive (Wittbrodt & Millard‐Stafford, 2018) performance may be attenuated in physically trained compared to untrained individuals. Repeated exposures to dehydration/hypohydration may also reduce its negative impact on endurance performance under temperate conditions (22°C) (Fleming & James, 2014), although such a possibility has not been replicated when exercise is performed under hot conditions (40°C) (Deshayes et al., 2021). Similarly, could heat acclimation improve the ability to better tolerate dehydration/hypohydration by reducing their adverse physiological effects? Some studies have reported that 10–21 days of active and traditional heat acclimation reduces the impact of pre‐exercise hypohydration (5% body mass loss) on thermal (Sawka et al., 1983) and cardiovascular (Buskirk et al., 1958; Sawka et al., 1983) strain during exercise in a temperate environment (i.e., 20–26°C), whereas heat acclimation only decreases cardiovascular strain (Sawka et al., 1983) during exercise in hot environments (35–49°C). Cheung and McLellan (1998) investigated whether 10 days of traditional and active heat acclimation attenuate the impact of pre‐exercise hypohydration (2.5% body mass loss) on a walking heat tolerance test to volitional fatigue while wearing protective equipment. They concluded that mild hypohydration impedes endurance capacity, but that heat acclimation does not attenuate this effect. However, a closer look at the data suggests that the negative impact of mild‐hypohydration on endurance capacity in the heat was slightly attenuated after heat acclimation in highly fit (pre: −12% vs. post: −4% compared with euhydrated) but not in moderately fit (pre: −19% vs. post: −21% compared with euhydrated) individuals. While this observation is interesting, it should be noted that some participants were forced to terminate the test due to reaching the ethical threshold for core temperature, which makes it impossible to know the effect on performance. More recently, Barley et al. (2020) reported that 12 days of passive and traditional heat acclimation may improve short‐duration repeat‐effort performance (sled‐push test) following ∼1% of body mass loss while also reducing perceptual strain (perceived exertion and fatigue). When assessed directly after 4% and ∼1% of body mass loss, cognitive performance, assessed using the CogState computerized test battery (simple and choice reaction time, learning and memory, and attention and working memory), was not different.

Overall, evidence remains scarce that heat acclimation reduces voluntary dehydration and its negative consequences on human physiology and functioning. Additional studies are needed to fill this gap, using more externally valid study designs that better reflect the conditions encountered in daily life situations (e.g., studying lower levels of dehydration, progressively induced through passive or active heat exposure rather than large levels of hypohydration induced before active heat stress).

### 4.3 Kidney function

Chapman et al. (2021) recently reviewed the kidneys’ integrative responses to acute and chronic heat stress. Readers are therefore referred to this comprehensive review for further details. Briefly, passive and, to a greater extent, active heat stress, acutely reduces renal blood flow, which may reduce glomerular filtration rate (GFR), although inconsistently observed in the literature, and increase the risk of acute kidney injuries (AKI). This phenomenon is exacerbated by mild dehydration/hypohydration (2.5% of body mass) in healthy young males and females (Chapman et al., 2023, 2020). This portrait is supported by studies showing an increased AKI prevalence during active heat stress in occupational (Moyce et al., 2016; Schlader et al., 2017; Schrier et al., 1970) and sports (Divine et al., 2018; Hodgson et al., 2017) settings, but also an increased risk of kidney injury during prolonged passive heat stress in healthy young males (8 h, 32–35°C, 95% humidity) (Hess et al., 2022). Thermal and cardiovascular adaptations inherent to heat acclimation, namely, improved body fluid regulation, expanded plasma volume, increased sodium reabsorption, and lowered core temperature during heat stress, suggest that heat acclimation may preserve kidney function and reduce the risk of AKI associated with heat stress. However, few studies have directly examined this possibility.

Only one study has investigated renal plasma flow (para‐aminohippurate clearance) during 90 min of rest, 90 min of moderate intensity cycling, and 150 min of rest in the heat (30°C, 60% relative humidity) before and after 4 days of active and traditional heat acclimation in healthy young and older males (Zappe et al., 1996). A reduced renal plasma flow during exercise and recovery was observed compared with resting values in both young and older males, and this response was unaffected by heat acclimation. How longer heat acclimation protocols, and using different approaches (e.g., controlled hyperthermia), may impact renal plasma flow remains to be further studied. Glomerular filtration rate has been studied after 7 days of passive and controlled hyperthermia heat acclimation (Ravanelli, Barry et al., 2021), 4 days of active and traditional heat acclimation (Zappe et al., 1996), 23 days of military heat acclimatization (Omassoli et al., 2019), 42 days of military summer training (Schrier et al., 1970), 10 days of heat acclimatization during a preseason football camp (Divine et al., 2018), and 4 days of active and controlled hyperthermia heat acclimation (Pryor et al., 2020). Except for the study by Ravanelli, Barry et al. (2021) that included five healthy females, all participants were healthy males. Among these studies, only Zappe et al. (1996) used the inulin clearance technique to estimate GRF, considered to be the gold standard. All other studies used the creatinine clearance technique or serum creatinine. Omassoli et al. (2019) reported that the acute decline in GFR that occurs after exercise heat stress is attenuated following heat acclimation. However, this result has not been replicated by more recent studies (Pryor et al., 2020, Ravanelli, Barry et al., 2021) that used short‐term passive or active controlled hyperthermia heat acclimation. Schrier et al. (1970) reported conflicting results, observing an improved creatinine clearance (indicative of improved GFR) following high‐intensity exercise from day 10 to 42 of military summer training in four males. However, they also reported in a larger sample (*n* = 12) that serum creatinine was similarly increased (indicative of a reduced GFR) after high‐intensity exercise between days 10 and 42 of the training program. Unfortunately, pre‐training values were not reported, making it impossible to know the full effect of heat acclimatization.

It is important to mention that an increased risk of kidney injury may occur even in the absence of reductions in kidney function (i.e., reductions in GFR and/or elevations in serum creatinine) (Hess et al., 2022). Few studies have investigated the effects of heat acclimation on AKI incidence during subsequent heat stress. Omassoli et al. (2019) and Pryor et al. (2020) reported that heat acclimation reduces the risk of developing AKI, according to clinical biomarker thresholds (i.e., serum creatinine and GFR) during moderate‐to‐high intensity exercise heat stress. In contrast, Haroutounian et al. (2021) demonstrated that 7 days of active and controlled hyperthermia heat acclimation with permissive dehydration (2–3% of body mass) does not increase biomarkers of AKI (urine neutrophil gelatinase‐associated lipocalin and kidney injury molecule‐1) in young healthy males. Interestingly, Pryor et al. (2020) observed that heat acclimation reduced the incidence of AKI without mitigating reductions in kidney function during intense exercise in the heat. These studies used changes in kidney function as diagnostic criteria for AKI, which is not without limitations (Chapman et al., 2021; Schlader et al., 2019). Therefore, investigating the risk of injury using biomarkers that reflect an increased susceptibility of the kidneys to AKI has been suggested (Chapman et al., 2021), and should be considered in future heat acclimation studies. Other pathophysiological markers, such as proteinuria and albuminuria, have also been investigated, but with conflicting results. Ravanelli, Barry et al. (2021) reported a reduction in the incidence of albuminuria during passive heat stress after 7 days of passive and controlled hyperthermia heat acclimation, whereas Schrier et al. (1970) reported an increased prevalence of proteinuria at day 42 of summer military training compared to day 10.

## 5 HUMAN HEAT ACCLIMATION – TO INFINITY AND BEYOND

This section provides an overview of key unanswered questions to stimulate further research within this field, with an emphasis on how such research will help humans thrive (i.e., work/sports performance) and survive (i.e., health) in a hotter climate. Based on the literature review presented, we propose the following areas of future research.

(1) It is well‐recognized that behavioural and physiological mechanisms contribute to body temperature regulation during heat stress (Schlader & Vargas, 2019). The former depends on voluntary decisions to reduce heat stress, and the latter is governed by a sequence of coordinated autonomic responses (Cramer et al., 2022). To date, most heat acclimation studies have focused on autonomic physiological adaptations and few studies have attempted to understand the effect on behavioural thermoregulation. More efforts are needed in this direction (Vargas et al., 2023).
(2) While heat stress can precipitate adverse health outcomes, it is worth recognizing that several factors, individual or related to the living environment, can modulate the sensitivity of individuals to heat stress and act as risk or protective factors (Ebi et al., 2021; Hajat et al., 2010). Thus, the fact that most of the literature was carried out on young (mostly male), healthy individuals, sometimes trained and having good cardiorespiratory fitness, which are all protective factors, strongly reduces the applicability of this evidence to the broader population. There is a need to better understand how various groups acclimate to heat, such as females, older adults, and people with chronic health conditions. For example, limited data suggest that sex and age influence the acquisition of heat acclimation adaptations (Notley et al., 2020; Wickham et al., 2021). While there is a general understanding regarding the temporal dynamic of heat acclimation induction (Périard et al., 2015), there is a need to improve our understanding of individual variability and better study how genetic factors, fitness levels, age, sex, and pre‐existing health conditions, among others, may influence heat acclimation induction and decay.
(3) To date, the available literature that considered the effects of heat acclimation on cognitive performance, voluntary fluid intake, and kidney function does not allow strong conclusions to be drawn. Further studies are needed to better understand the impact of heat acclimation on these parameters, using various protocols and populations. Furthermore, to improve our understanding of how heat acclimation might help maintain health despite more frequent heat exposures, more studies that investigate other health markers are needed. For example, investigating the potential effects of heat acclimation on sleep patterns (quality/quantity) and mental health could be important, as sleep disruption (Minor et al., 2022) and mental illness (Thompson et al., 2023) often accompany exposure to high temperatures.
(4) To improve our understanding of how to optimize health and performance under heat stress, future studies should continue efforts to refine, develop, and optimize heat acclimation protocols to make them more practical and applicable for at‐risk groups, especially those who have a reduced exercise capacity (Bach et al., 2024; Cole et al., 2023). There is also a need to explore how heat acclimation interacts with other interventions, such as common medication and supplements.
(5) In seasonal climates, heat acclimatization progressively develops across the summer months (Brown et al., 2022), conditional to adequate exposure to outdoor environmental heat. Conversely, household air conditioning has substantially increased in the last decades and humans live, work, and travel in air‐conditioned environments. It remains to be tested whether frequent air conditioning use may mitigate the acquisition and the decay of adaptations conferred by heat acclimation/acclimatization (Bain & Jay, 2011; Yu et al., 2012).

## AUTHOR CONTRIBUTIONS

Thomas A. Deshayes and Daniel Gagnon conceived and designed research; Thomas A. Deshayes and Daniel Gagnon prepared figures; Thomas A. Deshayes, Dèwanou Gilles Arnaud Sodabi, Marianne Dubord, and Daniel Gagnon drafted the manuscript. All authors have read and approved the final version of this manuscript and agree to be accountable for all aspects of the work in ensuring that questions related to the accuracy or integrity of any part of the work are appropriately investigated and resolved. All persons designated as authors qualify for authorship, and all those who qualify for authorship are listed.

## CONFLICT OF INTEREST

The authors have no conflicts to disclose.
